# Idiopathic Posterior Reversible Encephalopathy Syndrome (PRES) in a 4-month-old infant: a case report

**DOI:** 10.1186/s12883-026-05015-z

**Published:** 2026-06-26

**Authors:** Mehdi Oudrhiri Safiani, Hamza Zarouali, Saad El Harrak, Laarbi Ed Dafali, Hicham Ziani, Meryem Ennafiri, Aziza Bentalha, Salma Ech Cherif El Kettani

**Affiliations:** Pediatric Intensive Care and Anesthesiology Department, Children’s Hospital of Rabat, CHU Ibn Sina, Faculty of Medicine and Pharmacy of Rabat, Mohammed V University of Rabat, Rabat, Morocco

**Keywords:** Posterior Reversible Encephalopathy Syndrome (PRES), 4 months, Seizure, Idiopathic, Case Report

## Abstract

**Background:**

Posterior Reversible Encephalopathy Syndrome (PRES) is a clinico-radiological condition characterized by acute neurological symptoms associated with vasogenic cerebral edema. Although increasingly recognized in pediatric populations, PRES remains exceptionally rare in infants younger than six months, making diagnosis particularly challenging in this age group.

**Case presentation:**

We report the case of a 4-month-old previously healthy infant admitted for recurrent afebrile generalized tonic–clonic seizures. Initial clinical examination, laboratory investigations, cerebrospinal fluid analysis, and cranial computed tomography were unremarkable. Brain magnetic resonance imaging revealed bilateral, symmetrical cortico-subcortical occipital hyperintensities on T2-weighted and FLAIR sequences, with diffusion-weighted hyperintensity and mildly reduced ADC signal, overall suggestive of PRES with a possible superimposed cytotoxic component. No sustained arterial hypertension or underlying metabolic, infectious, autoimmune, or toxic cause was identified despite extensive evaluation, supporting the diagnosis of idiopathic PRES. Seizures were controlled with antiepileptic therapy, and the clinical course was favorable without early recurrence.

**Conclusion:**

This case highlights an exceptionally early presentation of idiopathic PRES in infancy and underscores the need to consider this diagnosis in unexplained seizures, even in the absence of classical risk factors. Early MRI is essential for diagnosis, and long-term neurodevelopmental follow-up remains crucial given the uncertain prognosis in this age group.

## Introduction

Posterior Reversible Encephalopathy Syndrome (PRES) is a clinico-radiological entity characterized by acute neurological symptoms [1] associated with predominantly vasogenic cerebral edema, resulting from impaired cerebral autoregulation and endothelial dysfunction. It typically presents with seizures, visual disturbances, altered consciousness, and characteristic bilateral parieto-occipital imaging abnormalities [2].

Although PRES is increasingly recognized in pediatric populations [3–5], it remains uncommon in children and exceptionally rare in early infancy. In this age group, diagnosis is particularly challenging due to non-specific clinical presentations, limited ability to assess visual or cognitive symptoms, and the broad differential diagnosis of seizures.

Only a limited number of cases have been reported in infants younger than six months [6–8], and idiopathic presentations without identifiable risk factors are even more exceptional. This highlights a gap in current understanding of age-specific pathophysiology and clinical expression of PRES in early life.

We herein describe an idiopathic case of PRES in a 4-month-old infant admitted for recurrent seizures.

## Case report

We present the case of a 4-month-old male infant with no significant medical history. Pregnancy was full-term and medically monitored, with no reported exposure to medications, toxins, or infectious diseases. Delivery was vaginal and uncomplicated, with immediate neonatal crying. No neonatal infections were reported. Apgar scores were 10 at 1, 3, 5, and 10 min. Psychomotor development was appropriate for age, including adequate head control. He was the youngest of four siblings, all in good health. The mother (G5P4) reported no consanguinity or notable familial medical conditions.

Symptoms began on the day of admission, with recurrent spontaneous generalized tonic-clonic seizures in an afebrile context. Between episodes, the infant had postictal recovery and preserved consciousness. This presentation prompted an emergency department consultation. On arrival, neurological examination showed a conscious, interactive infant without sensory or motor deficits. Pupillary responses were normal, and no seizure activity was observed during the assessment. There were no signs of raised intracranial pressure, including bulging fontanelle or projectile vomiting. Respiratory examination revealed eupnea with an oxygen saturation of 97% on room air and clear auscultation. Hemodynamic status was stable (blood pressure 100/70 mmHg; heart rate 120 bpm; warm extremities). The infant was afebrile (36.5 °C), and capillary blood glucose was 1.9 g/L. Muscle tone, including the lower limbs, was normal.

The infant was admitted to the pediatric neurology unit for further evaluation. Initial cranial computed tomography (CT) was normal. Additional seizure episodes occurred, resolving spontaneously or after midazolam administration (0.15 mg/kg), with complete recovery between episodes. Phenobarbital (20 mg/kg) was initiated, and a nasogastric tube was placed for airway protection during convulsions. Given concern for impending status epilepticus, the patient was transferred to the pediatric intensive care unit (PICU) for close monitoring and management.

Upon PICU admission, the patient was actively seizing, with perioral cyanosis and oral frothing. Heart rate increased to 200 beats per minute, and blood pressure transiently rose to 120/70 mmHg, with warm extremities. The infant remained afebrile (36.5 °C), and capillary blood glucose was 1.7 g/L. A bolus of midazolam (0.15 mg/kg) resulted in rapid seizure cessation, normalization of heart rate to 140 bpm, and full recovery of consciousness. Following seizure cessation and clinical stabilization, blood pressure returned to values within the normal range for age (systolic < 110 mmHg), with no further episodes of hypertension recorded throughout the remainder of the PICU stay.

Continuous non-invasive monitoring was instituted and peripheral venous access was secured. Phenobarbital was continued, and sodium valproate was introduced as second-line antiepileptic therapy, with strict hepatic monitoring given the patient’s age. Although sodium valproate is generally used with caution in infants under two years of age due to the risk of hepatotoxicity, it was considered the most appropriate second-line agent in this context given the refractory nature of the seizures, the absence of a suitable alternative for generalized seizure control, and the availability of close hepatic monitoring in the PICU setting. No further seizures occurred after initiation of dual therapy.

**Fig. 1:**
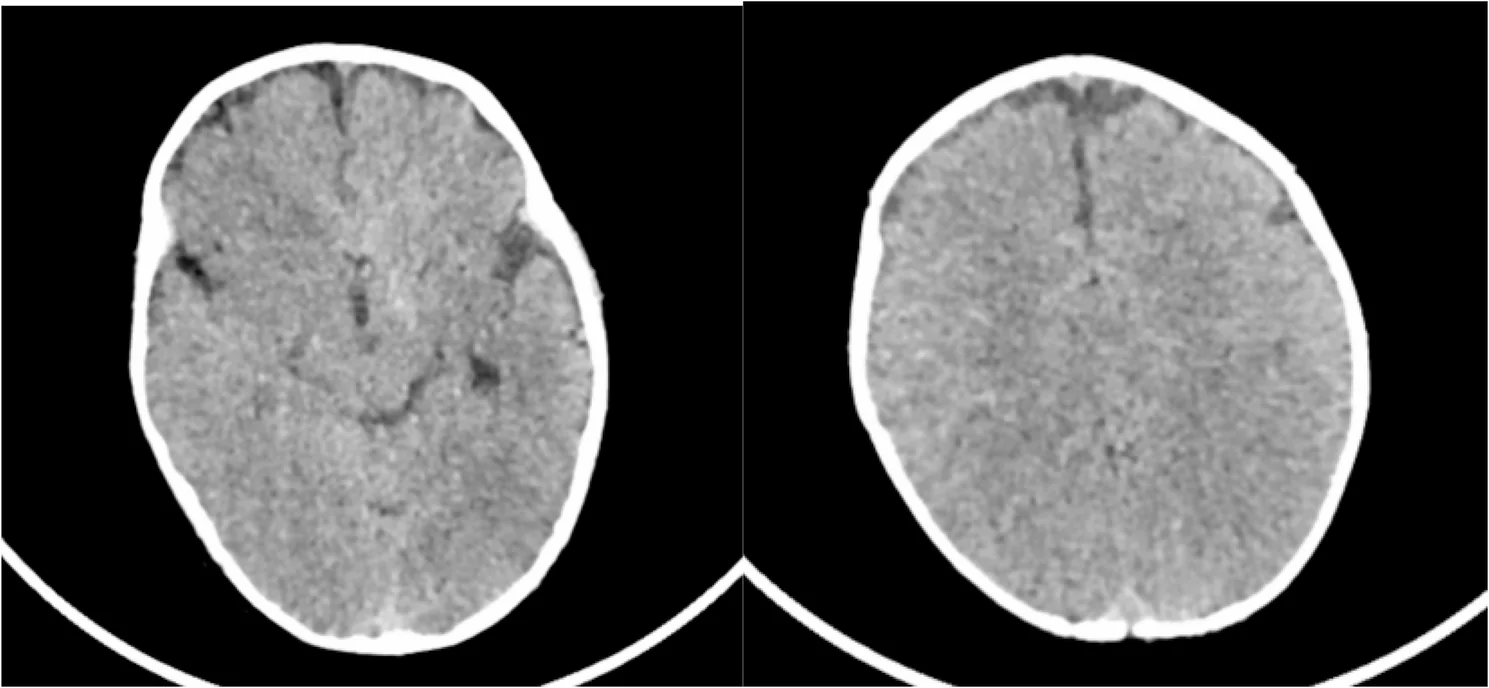
Axial brain CT scan revealing no parenchymal lesions or extra-axial collections

A repeat cranial CT scan remained normal (Fig. 1).

**Fig. 2:**
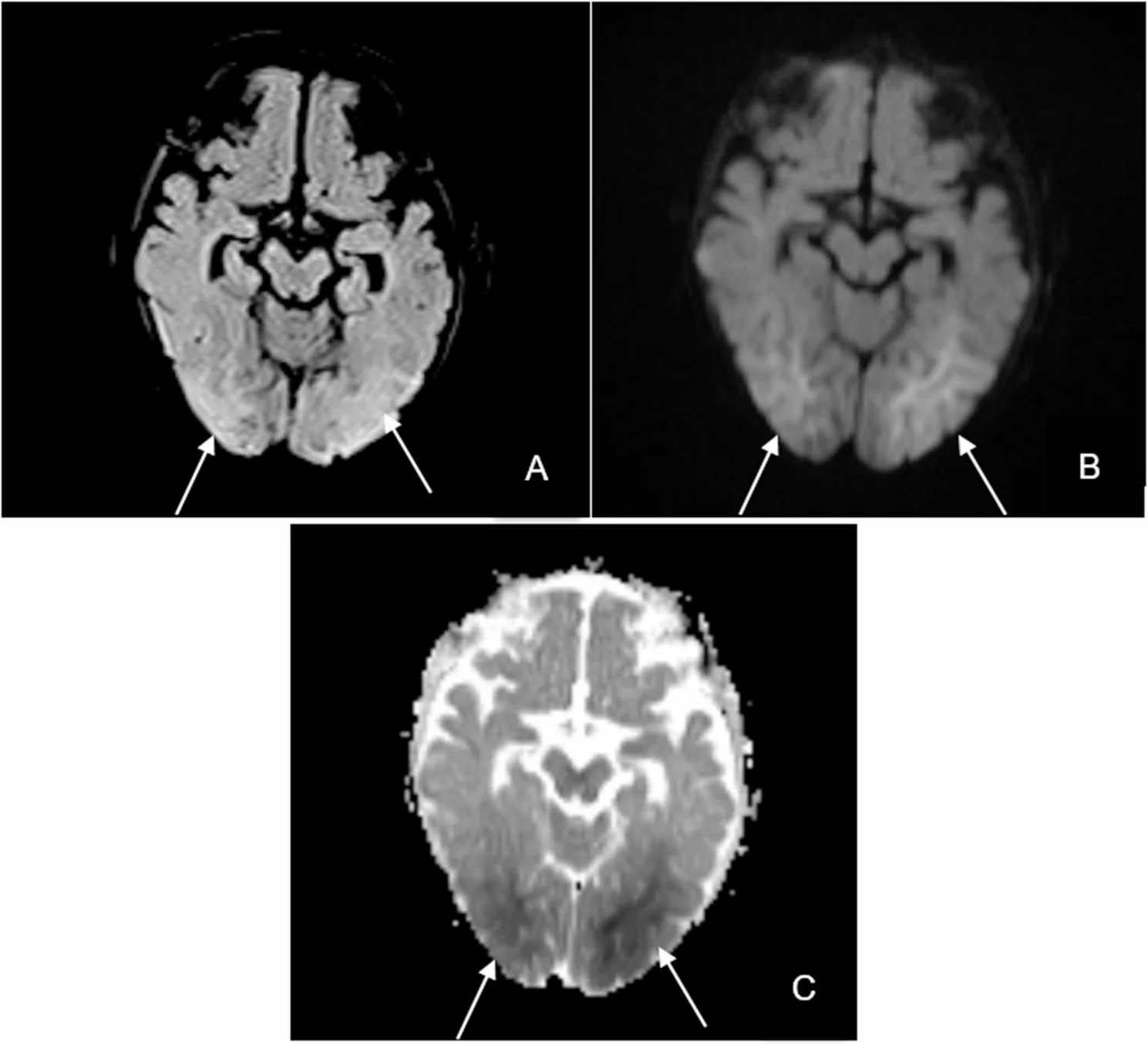
MRI identified a cortico-subcortical signal abnormality in the occipital lobes (thin arrows), bilaterally distributed with close symmetry. The lesions appear hyperintense on FLAIR (**A**) and diffusion-weighted imaging (**B**), with corresponding mildly reduced ADC signal on the ADC map (**C**). Overall, these findings are suggestive of PRES, predominantly vasogenic in pattern, with a possible superimposed cytotoxic component

Lumbar puncture showed normal cerebrospinal fluid findings. Given persistent diagnostic uncertainty, brain magnetic resonance imaging (MRI) was performed and revealed features characteristic of Posterior Reversible Encephalopathy Syndrome (Fig. 2).

MRI findings were suggestive of predominantly vasogenic edema, with a possible superimposed cytotoxic component, occasionally described within the PRES spectrum.

A comprehensive etiological assessment for PRES was undertaken, including metabolic, hepatic, and renal panels, as well as a complete blood count to exclude leukemia. All investigations were normal. There was no evidence of nephrotic syndrome or an infectious etiology. Blood pressure monitoring did not reveal sustained arterial hypertension outside seizure episodes. HIV, HBV, and HCV serologies were negative, as were autoimmune markers including p-ANCA, ANA, and others. Toxicology screening was negative for exogenous substances.

Interictal electroencephalography performed 48 h after stabilization showed no epileptiform discharges.

The diagnosis of PRES was considered based on the characteristic clinical and radiological features after exclusion of alternative diagnoses. The clinical course was favorable under treatment, with no recurrence of seizures. The patient was transferred back to the pediatric neurology unit for continued care. Short-term follow-up at one month showed persistent clinical stability (Table 1).

**Table 1:** Timeline of clinical events

Time point	Events
Day 0 – Morning	Sudden onset of recurrent spontaneous generalized tonic–clonic seizures in an afebrile context, with preserved consciousness and postictal recovery between episodes.
Day 0 – ED admission	Neurological examination: conscious, interactive infant, no focal deficits. Normal pupils. No signs of raised intracranial pressure. Stable respiratory and hemodynamic status (BP 100/70 mmHg, HR 120 bpm, SpO₂ 97% on room air). Afebrile (36.5 °C). Capillary blood glucose 1.9 g/L.
Day 0 – Neurology unit	Admission for evaluation. Initial cranial CT scan normal. Recurrent seizures occurred, resolving spontaneously or after midazolam (0.15 mg/kg). Phenobarbital loading dose (20 mg/kg) initiated.
Day 0 – PICU admission	Transfer due to concern for impending status epilepticus. Active seizure with perioral cyanosis and frothing. Transient tachycardia (HR 200 bpm) and blood pressure elevation (120/70 mmHg). Midazolam bolus (0.15 mg/kg) led to rapid seizure cessation and full recovery. Continuous monitoring established.
Day 0–1	Phenobarbital continued. Sodium valproate introduced as second-line antiepileptic therapy with close hepatic monitoring. No further seizures observed.
Day 1	Repeat cranial CT scan normal. Lumbar puncture revealed normal cerebrospinal fluid findings.
Day 2	Brain MRI performed due to diagnostic uncertainty: bilateral symmetrical cortico-subcortical occipital hyperintensities on T2 and FLAIR sequences, with corresponding mildly reduced ADC values, suggesting a possible cytotoxic edema component within the PRES spectrum.
Day 2–3	Extensive etiological workup: metabolic, hepatic, renal, hematological, infectious, autoimmune, and toxicological investigations all normal. No sustained hypertension outside seizure episodes.
Day 3	Interictal EEG (48 h after stabilization) showed no epileptiform discharges.

However, given the very early age of presentation, long-term neurodevelopmental prognosis remains uncertain. In particular, occipital involvement may be associated with delayed visual or cognitive deficits that may not become clinically apparent until later in childhood. Long-term follow-up is therefore essential to assess visual function, neurodevelopmental outcomes, and the potential risk of subsequent epilepsy.

## Discussion

PRES is a clinico-radiological syndrome presenting with non-specific neurological symptoms—such as headache, visual disturbances, focal deficits, seizures, and altered consciousness—accompanied by characteristic imaging findings [1, 2]. While well described in adults and older children, it remains exceptionally rare in early infancy [6–9].

The pathophysiology of PRES remains incompletely understood and involves a combination of impaired cerebral autoregulation [10, 11] and endothelial dysfunction [12], leading to vasogenic edema. These mechanisms may coexist, with varying contributions depending on the clinical context [13].

In pediatric patients, PRES is most commonly associated with renal disease, severe hypertension, immunosuppressive or chemotherapeutic agents, autoimmune disorders, and severe infections [14]. However, normotensive PRES has been increasingly reported in children, suggesting that endothelial dysfunction alone may be sufficient to induce edema even in the absence of sustained hypertension [15].

In the present case, the documented blood pressure elevation occurred exclusively during active seizures, suggesting a seizure-related sympathetic surge rather than sustained arterial hypertension. Whether seizures were the primary trigger of PRES through acute hemodynamic fluctuations, or an early manifestation of evolving vasogenic edema, cannot be formally determined and likely reflects a bidirectional interaction. In early infancy, immaturity of cerebrovascular autoregulation and reduced sympathetic innervation of the posterior circulation may facilitate blood–brain barrier disruption even during short-lasting hemodynamic fluctuations [15].

Seizures are the most frequent presenting manifestation of pediatric PRES [3–5]. Visual symptoms and encephalopathy may be difficult to assess in very young infants, potentially delaying diagnosis.

MRI remains the gold standard for diagnosis, typically revealing bilateral, symmetric parieto-occipital hyperintensities on T2-weighted and FLAIR sequences, reflecting vasogenic edema [16]. However, atypical imaging features may be observed, including diffusion restriction, hemorrhage, or contrast enhancement. In our case, diffusion-weighted hyperintensity associated with mildly reduced ADC signal suggested a possible cytotoxic edema component. Although this pattern is not the classic imaging presentation of PRES, it has been reported within the PRES spectrum, particularly in more severe or early-stage presentations, and does not preclude the diagnosis.

In the presence of reduced ADC signal suggesting a cytotoxic component, the main differential diagnoses include acute ischemic stroke, infectious encephalitis, metabolic encephalopathy, and mitochondrial disorders such as MELAS. However, these diagnoses were considered unlikely in our patient. Acute ischemic stroke was deemed improbable because of the absence of focal neurological deficits and the bilateral, symmetrical lesion distribution outside a vascular territory. Infectious encephalitis was considered unlikely given the normal cerebrospinal fluid findings and absence of fever. Metabolic and mitochondrial disorders were not supported by metabolic investigations or by the favorable short-term clinical evolution. Overall, the clinical-radiological presentation was more consistent with PRES than with alternative diagnoses associated with true irreversible cytotoxic injury.

The diagnosis of PRES in this case was based on the combination of typical clinical presentation and characteristic MRI findings, after exclusion of alternative diagnoses such as infectious encephalitis, metabolic disorders, and primary epileptic syndromes. The absence of epileptiform discharges on interictal EEG argued against an underlying primary epileptic syndrome as the cause of the seizures, further supporting the diagnosis of PRES.

According to published pediatric PRES causality frameworks, our case is consistent with previously proposed definitions of idiopathic PRES after exclusion of hypertensive, renal, infectious, toxic, metabolic, and autoimmune etiologies. Although follow-up MRI was not available in our patient, which limits formal demonstration of radiological reversibility, the overall clinical-radiological presentation and favorable short-term evolution strongly supported the diagnosis of PRES.

A limited number of PRES cases have been reported in early infancy. Kummer et al. [6] and Mrelashvili et al. [7] described PRES in infants, while Ohashi et al. [8] reported a central variant associated with mid-aortic syndrome. In contrast to these cases, our patient had no identifiable underlying condition despite an extensive etiological workup, supporting the diagnosis of idiopathic PRES. This highlights that PRES may occur even in the absence of classical risk factors in very young infants.

This case provides clinically relevant insights and highlights the occurrence of idiopathic PRES in very early infancy, a presentation rarely reported in the literature. First, it demonstrates that PRES can occur in very early infancy even in the absence of traditional risk factors such as hypertension, renal disease, or exposure to immunosuppressive agents. Second, it highlights that seizures may be the predominant or sole presenting feature in this age group, where visual and cognitive symptoms are difficult to assess. Third, it underscores the importance of early MRI in infants with unexplained seizures, as initial CT imaging may be normal. Finally, it emphasizes the need for cautious interpretation of short-term outcomes and systematic long-term neurodevelopmental follow-up.

Despite its name, PRES is not invariably reversible; neurological sequelae and death have been reported [17]. The term “potentially reversible encephalopathy syndrome” has been proposed to reflect this variability [18]. In our case, the short-term clinical outcome was favorable, with no recurrence of seizures at one-month follow-up. However, given the very early age at presentation, long-term neurodevelopmental prognosis remains uncertain.

Management of PRES in pediatric patients primarily relies on rapid seizure control, close hemodynamic monitoring, and identification of potential underlying triggers. In infants, particular attention should be given to airway protection during seizures and cautious use of antiepileptic drugs, considering age-specific pharmacological constraints.

Unlike adult populations, pediatric PRES may occur in the absence of sustained hypertension, highlighting the importance of mechanisms such as endothelial dysfunction. In our case, the absence of an identifiable trigger and the favorable response to antiepileptic therapy support a conservative, symptom-oriented approach.

Current pediatric literature emphasizes that early recognition and supportive management are key determinants of favorable outcomes, although evidence remains limited due to the rarity of PRES in this age group.

Overall, this case further expands the limited literature on PRES in early infancy and contributes to a better understanding of idiopathic presentations in this age group.

## Conclusion

Posterior Reversible Encephalopathy Syndrome (PRES) is a rare and complex clinico-radiological entity. This case highlights an exceptionally early idiopathic presentation in infancy, where diagnosis is particularly challenging because of non-specific clinical manifestations and the broad differential diagnosis of seizures. It also illustrates that diffusion abnormalities, including mildly reduced ADC signal suggesting a possible cytotoxic component, may be encountered and should not automatically exclude PRES when the overall clinical-radiological pattern is consistent. Early MRI is crucial, and prolonged neurodevelopmental follow-up is essential to detect delayed neurological or visual sequelae.

## Data Availability

All data generated or analyzed during this study are included in this published article. Due to the nature of this case report and to preserve patient confidentiality, no publicly available dataset has been created.

## Abbreviations

ADC: Apparent diffusion coefficient
ANA: Antinuclear antibodies
ANCA: Antineutrophil cytoplasmic antibodies
CT: Computed tomography
DWI: Diffusion-weighted imaging
FLAIR: Fluid-attenuated inversion recovery
HBV: Hepatitis B virus
HCV: Hepatitis C virus
HIV: Human immunodeficiency virus
MRI: Magnetic resonance imaging
PICU: Pediatric intensive care unit
PRES: Posterior reversible encephalopathy syndrome

## References

[1] Chaudhuri J, et al. Posterior Reversible Leucoencephalopathy Syndrome: Case Series, Comments, and Diagnostic Dilemma. Curr Neurol Neurosci Rep vol. 2023;23(8):433–49. 10.1007/s11910-023-01281-3 37378723

[2] Liman TG, Bohner G, Heuschmann PU, Endres M, Siebert E. The clinical and radiological spectrum of posterior reversible encephalopathy syndrome: the retrospective Berlin PRES study. J Neurol. 2012;259(1):155–64. 21717193 10.1007/s00415-011-6152-4

[3] Cordelli D, Maria, et al. Posterior reversible encephalopathy syndrome in infants and young children. Eur J Pediatr Neurol. 2021;30:128–33. 10.1016/j.ejpn.2020.10.009 33139147

[4] Habetz K, et al. Posterior reversible encephalopathy syndrome: a comparative study of pediatric versus adult patients. Pediatr Neurol. 2016;65:45–51. 27720711 10.1016/j.pediatrneurol.2016.09.001

[5] Chen T-H. Childhood posterior reversible encephalopathy syndrome: clinicoradiological characteristics, managements, and outcome. Front Pead. 2020;8:585. 10.3389/fped.2020.00585 PMC7518237 33042923

[6] Kummer S, Schaper J, Mayatepek E. D Tibussek.Posterior reversible encephalopathy syndrome in early infancy. Klin Padiatr. 2010;222(4):269–70. 20514606 10.1055/s-0030-1249031

[7] Mrelashvili A, Watson RE, Wong-Kisiel LC. Posterior reversible encephalopathy syndrome in an infant. Pediatr Neurol. 2013;49(5):387–8. 23993833 10.1016/j.pediatrneurol.2013.06.020

[8] Ohashi E, Hayakawa I, Tsutsumi Y, Kamei K, Ide K, Abe Y. Central-variant posterior reversible encephalopathy syndrome in an infant with mid-aortic syndrome: A rare case of symmetric basal ganglia lesions. Radiol Case Rep. 2022;17(10):3475–80. 35912291 10.1016/j.radcr.2022.07.024 PMC9334930

[9] Cordelli DM, Marra C, Ciampoli L, et al. Posterior Reversible Encephalopathy Syndrome in infants and young children. Eur J Paediatr Neurol. 2021;30:128–33. 10.1016/j.ejpn.2020.10.009. 33139147

[10] Bartynski WS. Posterior reversible encephalopathy syndrome, part 2: controversies surrounding pathophysiology of vasogenic edema. AJNR Am J Neuroradiol. 2008;29(6):1043–9. 18403560 10.3174/ajnr.A0929 PMC8118813

[11] MacKenzie ET, Strandgaard S, Graham DI, Jones JV, Harper AM, Farrar JK. Effects of acutely induced hypertension in cats on pial arteriolar caliber, local cerebral blood flow, and the blood-brain barrier. Circ Res. 1976;39(1):33–41. 1277403 10.1161/01.res.39.1.33

[12] Ando Y, et al. Posterior Reversible Encephalopathy Syndrome: A Review of the Literature. Intern Med (Tokyo Japan) vol. 2022;61(2):135–41. 10.2169/internalmedicine.7520-21 PMC8851194 34275982

[13] Marra A, Vargas M, Striano P, Del Guercio L, Buonanno P, Servillo G. Posterior reversible encephalopathy syndrome: the endothelial hypotheses. Med Hypotheses. 2014;82(5):619–22. 24613735 10.1016/j.mehy.2014.02.022

[14] Ghali MGZ, Davanzo J, Leo M, Rizk E. Posterior reversible encephalopathy syndrome in pediatric patients: pathophysiology, diagnosis, and management. Leuk Lymphoma. 2019;60(10):2365–72. 10.1080/10428194.2019.1594210. 31556774

[15] Ekinci F, Yildizdas D, Horoz OO, et al. Pediatric posterior reversible encephalopathy syndrome: Age related clinico-radiological profile and neurologic outcomes. Pediatr Int. 2023;65(1):e15562. 10.1111/ped.15562. 37310120

[16] Bartynski WS, Boardman JF. Distinct Imaging Patterns and Lesion Distribution in Posterior Reversible Encephalopathy Syndrome. Am J Neuroradiol 1 août. 2007;28(7):1320–7. 10.3174/ajnr.A0549 PMC7977645 17698535

[17] Burnett MM, Hess CP, Roberts JP, et al. Presentation of reversible posterior leukoencephalopathy syndrome in patients on calcineurin inhibitors. Clin Neurol Neurosurg. 2010;112:886–91. 20800343 10.1016/j.clineuro.2010.07.023

[18] Narbone MC, Musolino R, Granata F, et al. PRES: posterior or potentially reversible encephalopathy syndrome? Neurol Sci. 2006;27:187–9. 16897633 10.1007/s10072-006-0667-y

